# The Nutritional Quality Potential of Microgreens, Baby Leaves, and Adult Lettuce: An Underexploited Nutraceutical Source

**DOI:** 10.3390/foods11030423

**Published:** 2022-01-31

**Authors:** Eva Martínez-Ispizua, Ángeles Calatayud, José Ignacio Marsal, Claudio Cannata, Federico Basile, Abdelsattar Abdelkhalik, Salvador Soler, José Vicente Valcárcel, Mary-Rus Martínez-Cuenca

**Affiliations:** 1 Valencian Institute for Agricultural Research (IVIA), CV-315, Km 10.7, 46113 Valencia, Spain; martinez_evaisp@externos.gva.es (E.M.-I.); calatayud_ang@gva.es (Á.C.); marsal_jos@gva.es (J.I.M.); 2 Dipartimento di Agricoltura, Alimentazione e Ambiente (Di3A), University of Catania, Via Valdisavoia, 95123 Catania, Italy; claudio.cannata@phd.unict.it (C.C.); federico.basile@phd.unict.it (F.B.); 3 Horticulture Department, Faculty of Agriculture, Fayoum University, Fayoum 63514, Egypt; aga04@fayoum.edu.eg; 4 Valencian Institute for the Conservation and Improvement of Agrobiodiversity (COMAV), Polytechnic University of Valencia, Camino de Vera s/n, 46022 Valencia, Spain; salsoal@btc.upv.es (S.S.); jvalcarc@btc.upv.es (J.V.V.)

**Keywords:** antioxidant, biodiversity, baby leaf, landrace, lettuce, microgreen, mineral, nutraceutical compound

## Abstract

Interest in the cultivation of lettuce landraces is increasing because native varieties, as high-quality products, are particularly attractive to consumers. Lettuce is a popular leafy vegetable worldwide, and interest in the consumption of first leaves (microgreens) and seedlings (baby leaves) has grown due to the general belief that young plants offer higher nutritional value. The content of some bioactive compounds and antioxidants (chlorophylls, carotenoids, anthocyanins, ascorbic acid, phenols, antioxidant activity) was monitored in six lettuce landraces and five commercial varieties, and compared across three development stages: microgreen, baby, and adult. Ascorbic acid and phenolic contents were 42% and 79% higher, respectively, in the early stages than in adult lettuces, and red-leaf varieties (CL4 and L11) stood out. This finding agrees with lettuce’s marked antioxidant capacity and correlates with its pigment contents, especially anthocyanins. The nutritional value of adult lettuce is conditioned by its size, shape, and head structure as phytochemical concentrations are regulated by light. The low content of ascorbic acid, phenolics, and anthocyanins in crisphead lettuce (CL5) is a clear example (49, 67%, and 27% lower, respectively, than the adult mean). Our results indicate the wide variability of lettuces’ nutritional characteristics and emphasize that traditional varieties are a helpful source of agricultural biodiversity.

## 1. Introduction

Lettuce (*Lactuca sativa* L.) is a popular and widely grown leafy vegetable worldwide, especially as a component of salad mixes, whose consumption is increasing. Lettuce can contribute significantly to the nutritional content of diets [1]. In recent years, general consumer and researcher concern has been voiced about foods that, beyond nutritional needs, also provide health beneficial effects, for example, promote well-being, reduce diseases, and prolong life span. These effects are related to the nutritional quality of vegetables (minerals, vitamins, and phytochemicals with considerable antioxidant potential) [2,3]. 

The biosynthesis, composition, and concentration of health-promoting compounds varies widely among leafy vegetables, and are influenced by the genetic and environmental factors, growing conditions, harvest practices, and postharvest handling conditions [4]. As lettuce is generally eaten raw, more nutrients are preserved than in other cooked or processed vegetables, such as potatoes. Nevertheless, lettuce has not been regarded as a nutritional food, primarily because of its high water content (around 95%); however, its nutrient composition may be equivalent to other vegetables [5]. In lettuce, different plant attributes, such as leaf color, may influence the nutritional quality. One clear example is leaf pigmentation, which is often associated with the presence of antioxidant compounds. Red lettuce is highlighted for its lipophilic antioxidant activity and ascorbic acid and phenolic contents compared to other leafy vegetables (chicory, green lettuce, lamb’s lettuce, mizuna, red chard, red lettuce, rocket, spinach, Swiss chard, tatsoi), especially when exposed to low photosynthetically active radiation (PAR) light intensity. At high PAR, green lettuce has also been observed to have high contents of phenolic compounds [6]. In 11 lettuce cultivars, Lata and Przeradzka [7] determined that the antioxidant capacity provided by the glutamic acid and ascorbic acid contents was higher in the cultivars Kobra, Marion, and Red Bowl. Gazula et al. [8] worked with nine lettuce cultivars with differing numbers of genes to regulate carotene synthesis in them, and found that the highest pigment concentrations were found in the cultivars with the most genes in question. Comparisons of lettuces’ mineral contents are limited by the wide variation in the mineral contents reported in studies. This may be due to factors, such as different soil mineral compositions [9] and lettuce head types [10]. Studies have generally reported that lettuce is a relatively good source of Fe and little Na. Overall, among plant types, the mineral content was higher in butterhead, romaine, and leaf lettuces than in crisphead (iceberg) [11]. As lettuce is characterized by a marked ability to accumulate nitrate in the leaves, a low concentration is considered one of the most important healthy parameters, which is influenced by both genetic and environmental factors, especially light intensity [12].

Finally, plant age is interesting given the general belief that young plants have a higher nutritional value [13]. Consumption of first-development leaves (microgreens) to add texture and flavor to various dishes and salads consisting of seedlings (baby leaves) has gained popularity as a culinary trend [14,15]. This trend has been driven by two important market chain memberships: (1) growers, whose marketing strategy seeks to diversify the product offered and reduce cultivation periods to obtain higher profits; and (2) consumers, who are constantly searching for potential nutritional food and can make the most of microgreens’ easy at-home cultivation, especially as its availability in shops is scarce. So, given the popularity of lettuce worldwide, microgreens and baby types constitute a novel functional food that combines high sensory and bioactive values. This inspires comparisons with their mature-leaf counterparts, particularly as very few studies have examined their vitamin, nutrient, and carotenoid contents [9,15] and even fewer have provided comparative evidence of the phytochemical content of microgreens and baby leaves as opposed to their mature-leaf counterparts. The studies of Pinto et al. [9] and Weber [16] solely addressed the comparative mineral profiles of mature leaves and microgreens. El-Nakhe et al. [17] compared some nutraceutical compounds (chlorophylls, vitamin C, carotenes, phenolics), but this study was carried out with only two lettuce varieties at two harvest times (microgreen and adult). 

Another factor that induces variations in the nutritional quality of lettuce is the genetic material. Although there is compelling evidence for a declining nutritional value of horticultural crops, which is attributed to both changes in agricultural practices and the replacement of landraces with modern varieties and hybrids, promising new diet sources lie in local landraces, underutilized crops, and edible wild plants [18].

Hence, this study aimed to report the nutritional value of lettuce in relation to its different morphologies (color and head structure) and three harvest stages (microgreens, baby leaves, and adults) to determine the best health beneficial candidates that provide the highest nutritional value and bioactive compounds. Finally, it compared six Valencian lettuce landraces and five similar commercial varieties, and values, such as the usefulness of local varieties as a source of biodiversity.

## 2. Materials and Methods

### 2.1. Plant Material

**Figure 1:**
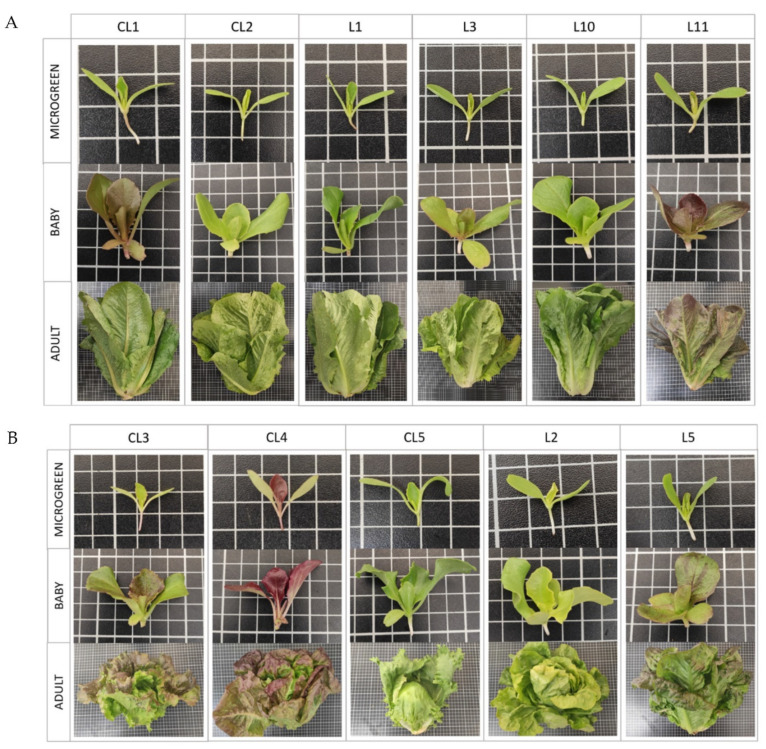
Pictures of the 11 cultivated lettuce varieties (*Lactuca sativa* L.) in the 3 development stages (microgreen, baby, adult) provided by the Germplasm Banks from the COMAV and the IVIA (Spain). The size of the grid cells in the fruit pictures is 0.01 m × 0.01 m. (**A**): Lettuce varieties without a patent head; (**B**): lettuce varieties with a prominent head.

The plant material for this study consisted of 11 lettuce *(Lactuca sativa* L.) varieties, including 6 landraces from the Valencian Community (Spain), which are diverse regarding their leaf color and head morphology. Seeds were provided by the Valencian Institute for the Conservation and Improvement of Agrobiodiversity (COMAV-UPV, Valencia, Spain) and the Valencian Institute of Agrarian Research (IVIA, Moncada, Spain). In addition, five commercial varieties were chosen as the most representative of the market formats. Table 1 provides the technical information of each variety. Figure 1A,B complement this table. 

### 2.2. Greenhouse-Field Experiments

Experiments were conducted from November to March in the IVIA experimental installations in Moncada (Valencia, Spain; 39°35′22.3″ N, 0°23′44.0″ W, 37 cm above sea level). Seeds were sown in November 2020 in 104-hole trays with 100% natural coconut coir fiber substrate (225 g L^−1^ density, Cocopeat, Projar Co., 46930 Quart de Poblet, Valencia, Spain) under greenhouse conditions (natural light conditions with a maximum PAR of 1000 µmol m^−2^ s^−1^, a mean temperature of 21 °C, and a mean humidity of 60%).

Two weeks after germination, the first group of seedlings (microgreen stage) was collected, after ensuring that the first true leaf had appeared, by cutting seedlings at the substrate level. Each microgreen sample comprised at least 20 seedlings, and each landrace or commercial variety consisted of 5 replicates.

A second group of plants was moved to an unheated greenhouse, where the temperature and light incidence were the same as in the external environment, thus preventing seedling thinning caused by high temperatures. Seedlings were protected from wind and potential pests while growing until the majority of the plants were 5 cm high (around 4 weeks after germination) after ensuring that at least 4 true leaves had appeared. One subgroup of seedlings was collected (baby stage) by cutting seedlings at the substrate level. Each landrace or commercial variety consisted of 4 replicates, with at least 10 seedlings each. The average range of the minimum and maximum temperatures was 4–26 °C for November and −1–26 °C for December. 

Finally, a second subgroup of seedlings was transplanted on 4 December 2020, and grown under field conditions. Each landrace or commercial variety consisted of 20 plants grown in 2 separate replicates (10 single plants each) cultivated in single rows (110 cm apart) with 30- and 60-cm spacings between each plant and variety, respectively. The plot was surrounded by border rows on all four sides. The soil composition within a depth of 20 cm was 68% sand, 11% clay, and 21% silt (sandy-clay loam), and contained 0.61% organic matter, 0.051% total N, less than 8 mg kg^–1^ P, 301 mg kg^–1^ K, and 2.87 meq·100 g^–1^ assimilable Mg. The soil electrical conductivity was 0.290 dS m^–1^ and pH was 8.1. 

Irrigation satisfied 100% crop evapotranspiration (ETc), as described in Penella et al. [19], performed with a drip system. Nutrients were applied by an irrigation system at a rate (kg ha^−1^) of 200 N, 50 P_2_O_5_, 250 K_2_O, 110 CaO, and 35 MgO, as recommended by Maroto [20]. The average range of the minimum and maximum temperatures during the field experiment was 1–23 °C for December, −1–26 °C for January, 7–24 °C for February, and 6–26 °C for March. Plants (adult stage) were harvested on 16 March. 

### 2.3. Leaf Sample Preparation

For the microgreens and baby material, 8 different replicates (2 g of vegetal material per replicate) of each variety were obtained by randomly grouping seedlings (around 20 and 10 plants per replicate for microgreens and baby, respectively). Four of these replicates were reserved for stove drying. The remaining fresh samples were instantly frozen in liquid nitrogen and stored at −80 °C. Of the adult plants, 4 different replicates (1 individual lettuce each replicate) were harvested from the field. Lettuce was cut lengthwise into four halves. A fraction of each lettuce was set aside for drying. A second fraction was chopped and instantly frozen in liquid nitrogen and stored at −80 °C. 

The plant material reserved for drying was used for the mineral analysis and dry weight (DW) quantification while the samples stored at −80 °C were employed for nutraceutical quality determinations. Samples were ground in a mixer mill (MM400, Retsch, Hann, Germany) with liquid nitrogen to prevent melting. The same machine was used to homogenize the samples dried in a laboratory oven at 65 °C for 72 h. 

### 2.4. Nutraceutical Compounds and Antioxidant Capacity

#### 2.4.1. Chlorophyll and Carotenoid Concentration

Total chlorophyll (Chl) a + b and carotenoids (Car) concentration were determined spectrophotometrically as described by Porra et al. [21]. Briefly, 2.5 mL of 80% acetone (*v*/*v*) were added to the sample extracts (0.06 g FW) and centrifuged at 2000 rpm for 8 min. The supernatant was used for the analysis. Solution absorption was measured at 663.6, 646.6, and 470 nm using a spectrophotometer (Lambda 25 UV/VIS, Perkin Elmer, Waltham, MA, USA). Then, 80% acetone (*v*/*v*) was utilized as the blank solution. The chlorophyll and carotenoid contents of the extracts were calculated by the following equations:

(1) Chl a = 12.25 × Abs663.6 − 2.79 × Abs646.6 (µg mL^−1^)
(2) Chl b = 21.3 × Abs646.6 − 5.1 × Abs663.6 (µg mL^−1^)
(3) Car = [(1000 × Abs470 − 1.82 Chl a) − (85.02 × Chl b)]/198 (µg mL^−1^)
(4) Chl a + b = 7.15 × Abs663.6 + 18.71 × 646.6

Chlorophylls and carotenoids were expressed as µg g^−1^ FW.

#### 2.4.2. Anthocyanin Concentration

The anthocyanin (Ant) concentration was spectrophotometrically quantified as described by Szepesi et al. [22]. In total, 5 mL of methanol:HCl:H_2_O solution (90:1:9) were added to 0.1 g of FW of the homogenized sample previously placed in glass tubes. Samples were vortexed and stored in the dark for 1 h. The samples in the tubes were mixed at room temperature. Then, they were centrifuged at 2000 rpm for 5 min and the supernatant was used for the analysis. Solution absorption was measured at 534, 643, and 661 nm using a spectrophotometer (Lambda 25 UV/VIS, Perkin Elmer, Waltham, USA). Methanol:HCl:H_2_O solution was employed as the blank. The Ant content of the extracts was calculated by the following equation:

(1) (0.0821 × Abs534 − 0.00687 × Abs643 − 0.002426 × Abs661) × 5 mL g^−1^ FW

The anthocyanin concentration was expressed as µmol 100 g^−1^ FW.

#### 2.4.3. Ascorbic Acid Concentration

The total ascorbic acid (AsA) content was spectrophotometrically quantified as described by Kampfenkel et al. [23]. First, 0.2 g FW of each homogenized sample were added to 1.5 mL of 6% (*w*/*v*) trichloroacetic acid (TCA). Samples were centrifuged at 15,000 rpm for 5 min at 4 ºC and the supernatant was recovered. Then, 0.05 mL of the homogenate were mixed with 0.05 mL of 10 mM dithiothreitol (DTT) and 0.1 mL of 0.2 M phosphate buffer (pH 7.4). Samples were incubated for 15 min at 42 °C. Next, 0.05 mL of 0.5% (*w*/*v*) N-ethylamide (NEM) were added and incubated for 1 min at room temperature. Afterwards, 0.25 mL of 10% (*w*/*v*) TCA, 0.2 mL of H3PO4 4% (*w*/*v*), 0.2 mL of 2-2′-dipyridyl, and 0.1 mL of 3% (*w*/*v*) FeCl3 were added to the solution. They were incubated together in a water bath for 40 min at 42 °C. The solution absorption was measured at 525 nm using a spectrophotometer (Lambda 25 UV/VIS, Perkin Elmer, Waltham, MA, USA). The blank solution with no extract was used for calibration. AsA was expressed as mg 100 g^−1^ FW.

#### 2.4.4. Total Phenolic Analysis

The phenolic (Phe) content was analyzed according to Dewanto et al. [24] with minor changes. Firstly, a 0.1 g FW aliquot of the homogenized sample was homogenized in 0.7 mL of 80% (*v*/*v*) methanol, vortexed, incubated in an ultrasonic bath (Ultrasonic cleaner, Fungilab, Barcelona, Spain) at medium intensity for 30 min, and then revortexed. Samples were centrifuged at 10,000 rpm for 15 min at 4 °C and the supernatant was reserved. The total Phe content was determined by the Folin–Ciocalteau colorimetric method. Following this, a 20 µL aliquot of the supernatant was mixed with 80 µL of methanol and 0.7 mL of Folin–Ciocalteau reagent. This solution was vortexed and incubated in the dark for 5 min at room temperature. Next 0.7 mL of NaHCO3 (6%) were added. The final solution was vortexed and incubated in the dark for 60 min. The solution absorption was measured at 765 nm in a spectrophotometer (Lambda 25 UV/VIS, Perkin Elmer, Waltham, MA, USA). Blank solution with no extract was used for calibration. Each measurement was compared to a standard curve of gallic acid (GA). The Phe concentration was expressed as mg of GA equivalent g^−1^ FW.

#### 2.4.5. Antioxidant Capacity Measurements

The antioxidant capacity (DPPH) was measured following the method reported by Brand-Williams et al. [25] with minor changes. Firstly, 0.1 g FW of sample were homogenized in 0.7 mL of 80% methanol (*v*/*v*), incubated in an ultrasonic bath (Ultrasonic cleaner, Fungilab, Barcelona, Spain) at medium intensity for 30 min, and then vortexed. Samples were centrifuged at 10,000 rpm for 15 min at 4 °C and 20 μL of the extract were added to 990 μL of 0.065 M of 2,2-diphenyl-1-picrylhydrazyl solution (solved in 80% methanol). The absorbance was measured at 515 nm against a blank solution (80% methanol without extract) after a 30-min reaction at room temperature in the dark using a spectrophotometer (Lambda 25 UV/VIS, Perkin Elmer, Waltham, USA). The results were expressed as the percentage reduction of the initial DPPH absorption in extracts.

### 2.5. Mineral Determination 

Samples were dried in a laboratory oven at 65 °C for 72 h and homogenized before being burnt in a muffle furnace for 12 h at 550 °C. Macronutrients and micronutrients were extracted with 5 mL of 2% (*v*/*v*) nitric acid in an ultrasonic bath for 30 min at 40 °C. Afterwards, 10 mL of 2% nitric acid were added to the solution. Mineral concentrations were measured by ICP emission spectrometry (iCAP 6000, Thermo Scientific, Cambridge, UK). The results for the macro- and micronutrients were expressed as mg g^−1^ DW and µg g^−1^ DW, respectively.

### 2.6. Statistical Analysis

The results obtained from these determinations were subjected to a one-way analysis of variance (ANOVA) using Statgraphics Centurion XVII (Statistical Graphics Corporation 2014). The statistical analysis was carried out after taking two different factors into account: the variety type and development stage. The results were expressed as mean ± standard deviation. Means were accepted as being significantly different at a 95% confidence interval (*p* ≤ 0.05). The mean, maximum and minimum values, coefficient of variation, and F-ratio of all the traits were calculated. 

A principal component analysis (PCA) was run for the standardized values using pairwise Euclidean distances among accession means to determine the relations between genotypes in each development stage. The extracted eigenvalues, and the relative and cumulative proportions of total variance explained by the first three principal components (PCs) were calculated. A two-dimensional (2D) scatter plot (first PC vs. second PC) for each development stage was prepared based on a distance matrix for the PCs to visualize the relation that explained traits.

By considering the quality traits, three correlation analyses were completed among the varieties, one for each development stage. The individual samples of each accession were subjected to linear regression and correlation coefficients (r) were obtained.

## 3. Results

### 3.1. Dry Weight

**Figure 2:**
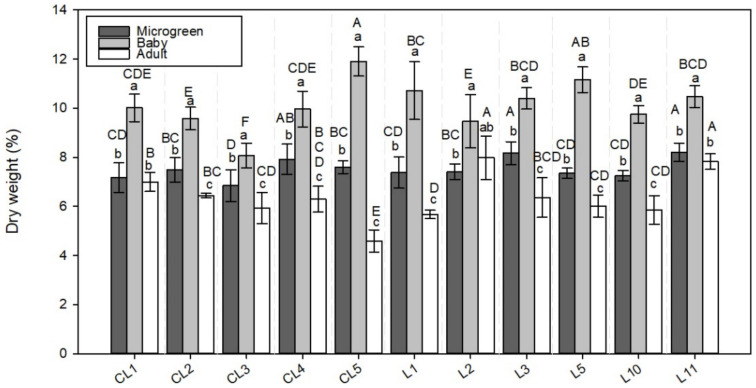
Dry weight (DW) in the 11 lettuce varieties evaluated in the 3 development stages (microgreen, baby, adult). Values are the mean ± SE of four replicates per landrace. The mean was subjected to a one-way ANOVA. Different capital and lowercase letters indicate significant differences between varieties and development stages, respectively, at *p* < 0.05 by the LSD test.

Three varieties (CL1, L2, L11) presented no statistical differences in % DW between the microgreen and adult plants (Figure 2). The highest values were recorded for CL4, L3, and L11 in the microgreen plants (nearly 0.7% higher than the mean value) and CL5 and L5 in the baby stage (1.8 and 1.1% higher than the mean value, respectively). L2 and L11 showed the highest DW percentage in the adult stage (2.3% and 1.3% over the mean value, respectively). CL3 and CL5 in the baby and adult stage, respectively, had the lowest percentage of dry biomass (nearly 2.0% lower than their mean values).

### 3.2. Nutraceutical Compounds and Antioxidant Capacity

#### 3.2.1. Total Chlorophyll Concentration

**Figure 3:**
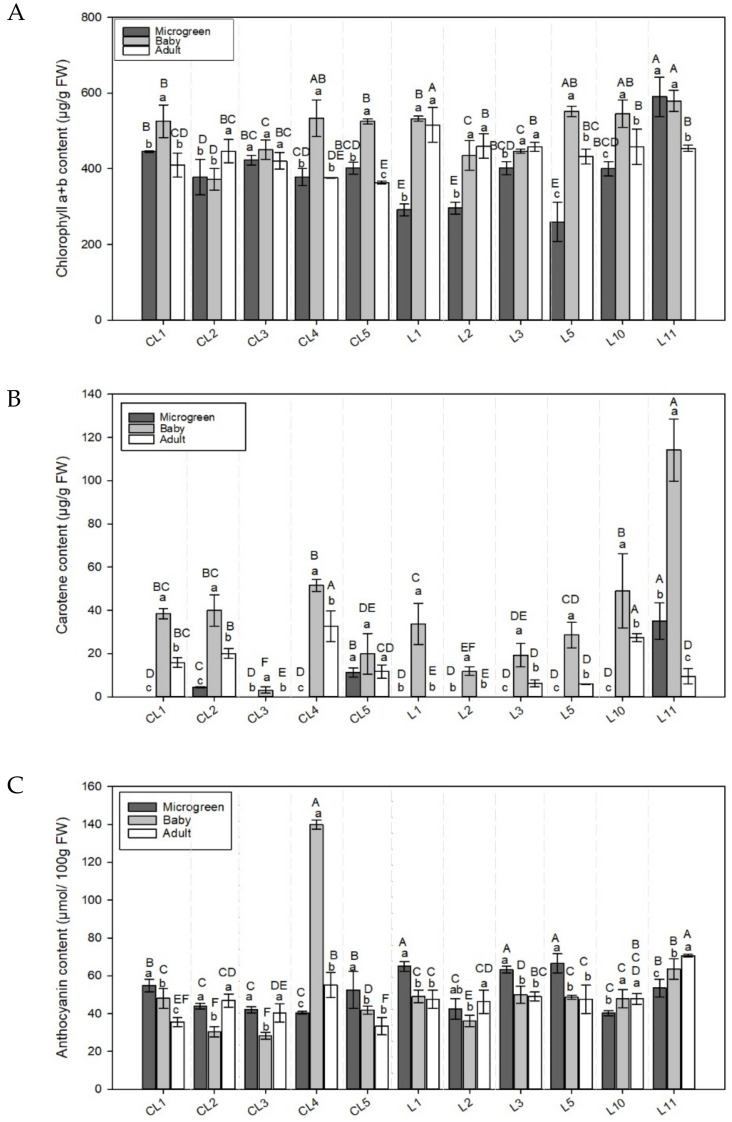
The (**A**) chlorophyll a + b (Chl), (**B**) carotenoid (Car), and (**C**) anthocyanin (Ant) concentrations in the 11 lettuce varieties evaluated in the 3 development stages (microgreen, baby, adult). Values are the mean ± SE of four replicates per landrace. The mean was subjected to a one-way ANOVA. Different capital and lowercase letters indicate significant differences between varieties and development stages, respectively, at *p* < 0.05 by the LSD test. FW: Fresh weight.

The highest chlorophyll content (Table 2) was recorded in the baby stage (mean value 27.7% and 15.8% higher than microgreens and adults, respectively) in all the varieties but CL2 (Figure 3A), with no significant differences with the adult stage in some varieties (CL3, L1, L2, L3). 

In the microgreen stage, landrace L11 had the highest Chl content (49.9% over the mean) while the lowest values were shown by L1, L2, and L5 (282.4 ± 19.9 µg g^−1^ FW; 23.9% under the mean value) (Figure 3A). 

The highest Chl levels in the baby plants were obtained in CL4, L5, L10, and L11 while CL2 had the lowest values. Adult lettuces L1 and CL5 were highlighted for their highest and lowest Chl contents, respectively.

#### 3.2.2. Carotenoids

Table 2 shows the Car content in the different development stages, which was higher in the baby stage than in the other development formats (mean values of 893.5% and 230.9% higher than the microgreens and adults, respectively).

In the microgreen stage (Figure 3B), 3 of the 11 varieties (CL2, CL5, L11) contained Car compounds, which were not detectable in the other varieties. In the baby stage, all the plants contained Car, which were remarkable in L11 (114.5 µg g^−1^ FW, 210.5% over the mean value) and also in L10 and 3 commercial varieties (CL1, CL2, CL4) for ranging between 38.5 and 48.9 µg g^−1^ FW. Of the adult lettuces, CL4 and L10 showed the highest Car level, which was not detectable in three varieties: CL3, L1, and L2.

#### 3.2.3. Anthocyanins

One detected trend was that the highest Ant content was observed in the commercial varieties and landraces in the microgreen stage (mean values of 50.7 and 56.2 µmol 100 g^−1^ FW, respectively, Table 2), expect in CL4, L10, and L11, which showed higher contents in the baby and adult stages (Figure 3C).

Of the microgreens, 3 local landraces (L1, L3, L5) stood out for their high (64.9 ± 1.7 µmol 100 g^−1^ FW, Figure 3C). In the baby stage, Ant levels were notably elevated in CL4 (148.9% higher than the mean value) and low in CL2 and CL3 (nearly 45% lower than the mean). Of all the adult plants, L11 had the highest Ant content (48.33% over the mean) and CL5 has the lowest content (29.94% under the mean).

#### 3.2.4. Ascorbic Acid

**Figure 4:**
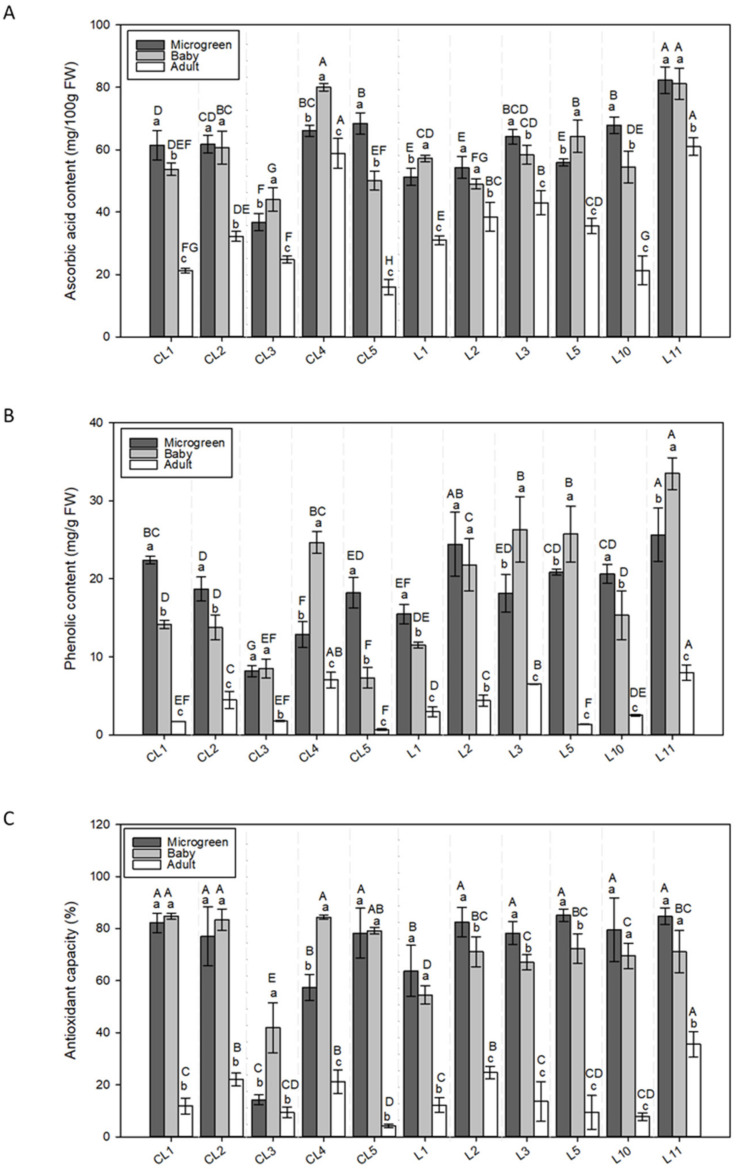
The (**A**) ascorbic acid (AsA), (**B**) phenols (Phe), and (**C**) antioxidant (DPPH) capacity of 11 lettuce varieties evaluated in 3 development stages (microgreen, baby, adult). Values are the mean ± SE of four replicates per landrace. The mean was subjected to a one-way ANOVA. Different capital and lowercase letters indicate significant differences between varieties and development stages, respectively, at *p* < 0.05 by the LSD test. FW: Fresh weight.

The maximum AsA concentration appeared in the microgreen and baby stages (Table 2), and L11 presented the highest AsA levels (34.8% and 39.1% higher than the mean value for both stages, respectively) (Figure 4A). CL4 also had a high AsA level in the baby stage. The AsA concentration in adult lettuce (mean value 34.45 mg 100 g^−1^ FW, Table 2) dropped in both the commercial and local landraces, with the lowest values shown for CL5 (53.8% lower than the mean). The highest AsA content in the adult stage was observed in lettuces CL4 and L11 (70.7% and 77.0% higher than the mean value, respectively).

#### 3.2.5. Phenols

The mean Phe content (Table 2) was similar in the microgreen and baby stages, and was around 79% higher than in adult lettuces. Two landraces (L2 and L11) stood out for their high Phe content in the microgreen stage (around 25.0 mg g^−1^ FW) (Figure 4B). In the baby stage, the most remarkable varieties were CL4 and local landraces L3 and L5, especially L11 (between 24.7 and 33.5 mg g^−1^ FW). CL3 had the lowest Phe content in both the microgreen and baby stages (56.3% and 53.9% lower than the mean, respectively). The highest Phe content in the adult stage was observed in the lettuces CL4 and L11 (79.8% and 104.1% higher than the mean value, respectively). In total, 3 of the 5 commercial varieties (CL1, CL3, CL5) showed very low Phe contents in the adult stage (from 0.7 to 1.8 mg g^−1^ FW), which occurred in only 1 local landrace: L5 with 1.4 mg g^−1^ FW.

#### 3.2.6. Antioxidant Capacity

Similar to the AsA and Phe contents, the greatest DPPH activities in the commercial varieties and landraces appeared in the microgreen and baby development stages (mean values of 71.5% and 71.1%, respectively, Table 2), expect in CL3, which was higher only in the baby stage. No significant differences between the microgreen and adult stages were found (Figure 4C). A lower antioxidant capacity was measured in the adult stage (around 55.6% lower than in the other two stages, Table 2).

When comparing the varieties across their different development formats, the highest DPPH levels in the microgreen lettuces were observed for 8 of the 11 varieties, except CL3, CL4, and L1 (Figure 4C). In this stage, most local landraces presented between 6.8% and 13.6% more DPPH activity than the mean (71.5%), and slightly lower activity was observed for L1 only (7.8%). 

In the baby stage, the lowest DPPH was displayed in CL3 and L1 (29.1% and 16.5% lower than the mean, respectively). In the adult stage (mean value 15.9%, Table 2), the greatest activity was observed in L11 (19.7% higher than the mean value), with a significant difference (*p* < 0.05).

### 3.3. Mineral Concentration

Table 3 shows the concentration of three of the main minerals (Ca, K, Fe) related to lettuce’s nutritional quality. The results for the other macro- and micronutrients are shown in Supplementary Table S1.

The maximum Ca concentration was found in the microgreen stage in all the varieties (Table 2) but L11, which was exceeded by adult lettuce. Of the varieties, 4 of the 6 local landraces (L2, L5, L10, L11) and 1 commercial lettuce (CL3) in the microgreen stage obtained the highest Ca concentrations (between 10.28 and 12.38 mg g−1 DW, Table 3) while the lowest value was shown by L1 (26.42% lower than mean value, Table 2). In the baby stage, the most remarkable varieties were CL3, CL4, L4, L5, L10, and L11 (between 7.76 and 8.89 mg g^−1^ DW). The Ca content in adults was also high in CL4 and two other local landraces (L1 and L3). 

As a general trend, the adult stage presented the highest K concentration, followed by microgreens, with the lowest levels observed in the baby stage (52.15% and 41.57% lower than the previous ones, respectively) (Table 2). The highest K concentrations in microgreens were obtained for CL3, CL5, L3, L5, and L10 (between 48.9 and 53.6 mg g^−1^ DW, Table 3). The highest K levels in the baby stage were observed in the varieties CL3, CL4, and L2 (between 30.3 and 32.5 mg g^−1^ DW). In the adult stage, 2 commercial varieties (CL2 and CL3) and 2 local landraces (L1 and L3) stood out for their high K contents (between 66.0 and 68.7 mg g^−1^ DW). 

Another trend that was observed was the lowest Fe concentrations, which were recorded in the baby stage (mean value 53.5% and 39.0% lower than the microgreen and adult stages, respectively, Table 2). Depending on the variety, the highest significant Fe levels (Table 3) were obtained in the microgreen stage (CL1, CL2, CL5, L2, and L3), adult stage (CL3, CL4, and L11), or both (L1, L5, and L10). Of the microgreens, the varieties L2 and CL3 presented the highest and lowest Fe concentrations (344.9 and 77.7 µg g^−1^ DW, respectively, Table 3). In the baby stage, the Fe concentration was more homogeneous (low CV%, Table 2) and 5 of the 11 varieties (CL1, CL3, CL4, L2, and L11) showed top levels (between 114.7 and 122.5 µg g^−1^ DW). The highest Fe concentration in adult lettuces was observed in local landrace L11 (187.8% higher than the mean adult value, Table 2). IIn total, 3 commercial varieties and 1 local variety (CL1, CL2, CL5, and L3) presented the lowest Fe level in the adult stage (between 50.8% and 35.8% lower than the mean).

### 3.4. PCA Analysis

The PCA and eigenvalues higher than 1 reflected a different pattern in the correlation of lettuces in the three development stages (Table 4). In all cases, there were 3 significant PCs that described around 83%, 75%, and 76% of the variability between varieties in the microgreen, baby, and adult stages, respectively. 

In microgreens, the first, second, and third PCs accounted for 46.9%, 22.8%, and 13.5% of the total variation of the studied traits, respectively. The first PC correlated positively with all the traits, except for a negative correlation with the K concentration (−0.409), and the AsA concentration had the highest value (0.419). When analyzing the second PC, the highest positive correlation was recorded for the Fe mineral (0.435), with negative correlations observed for Chl and Car (−0.557 and −0.294, respectively). 

In the baby stage, the relevance of the first PC was less than in the other 2 stages and accounted for only 34.6% of the total variation. The second and third PCs accounted for 26.4% and 13.9% of the variability, respectively. Regarding the correlation values in the first PC, all the traits were positively correlated, and the most significant results were obtained for the Car, Phe, and AsA concentrations. The highest positive correlations in the second PC corresponded to the mineral contents (Ca, K, and Fe) while DW was negatively correlated (−0.471). 

The distribution of the adult lettuces in the PCA was determined mostly by the variability of the traits in the first PC (50.9%) while the second and third PCs represented only 14.4% and 10.7% of the variation, respectively. Most traits presented a moderate positive correlation of the first PC, and DPPH, Phe, and AsA had the highest values (between 0.406 and 0.369). A moderate value was also obtained for the negative correlation (−0.360) with the Chl content of the first PC. When analyzing the second PC, the highest positive correlation was for observed for the Car concentration (0.593) while the most negative value was shown by the Ca content (−0.428). 

**Figure 5:**
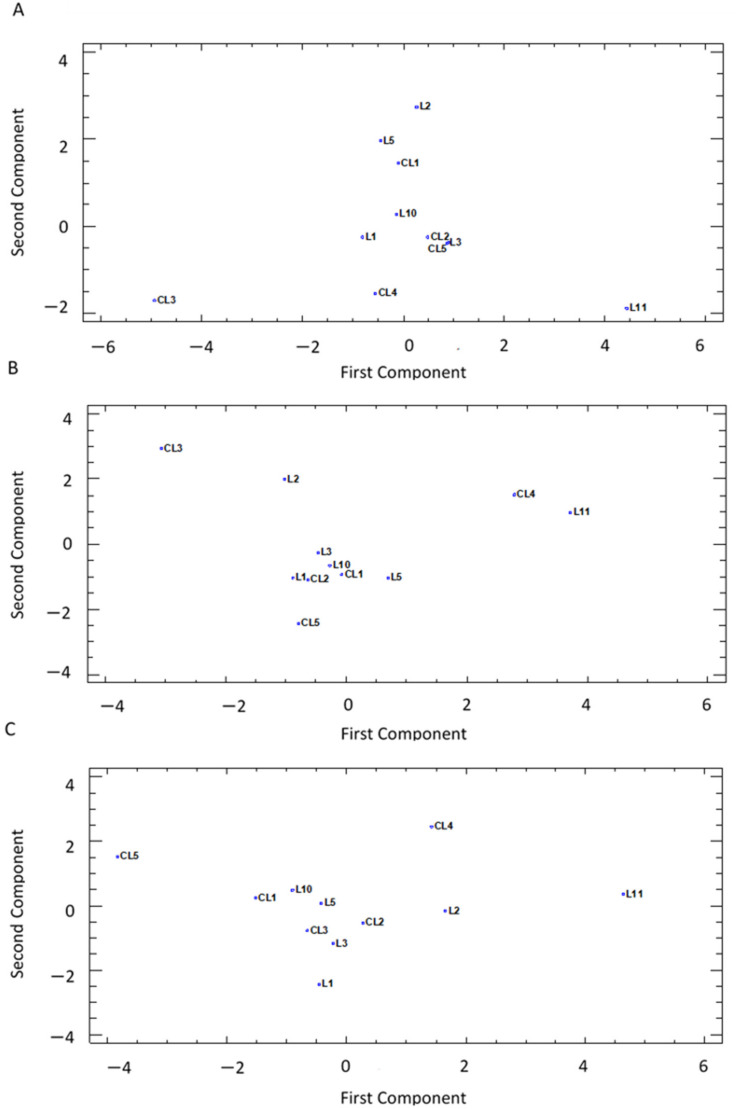
Principal component analysis (PCA) of 11 lettuce varieties based on quality traits represented in the 2 first components of the PCA for the (**A**) microgreen stage (46.99% and 22.80% of the Table 3 and Table 4. 60% and 26.43% of the total variation, respectively), and (**C**) adult stage (50.88% and 14.44% of the total variation, respectively).

For the three development stages, the projection on the PCA plot for the first and second PCs (Figure 5) showed a similar pattern of spread over the area. In general terms, there was a group with a large number of varieties located in the central zone of the graphs while two or three varieties were located further to the right or the left of the plots. 

In microgreens, the highest value was recorded for the first PC (right zone) for landrace L11 (Figure 5A) and its top levels correlated with four traits: Car, AsA, Phe, and DPPH (Figure 3B and Figure 4A–C) and low K levels (Table 3). On the contrary, the lowest values for AsA, DPPH, and Phe throughout the experiment meant the variety CL3 was located further to the left of the plot. For the second PC, local landrace L2 was located at the top of the plot due to its high Fe content (Table 3) and low Chl concentration (Figure 3A) while L11 was located at the bottom due to its high pigments concentration (Chl and Car, Figure 3A,B). The low Fe level in CL3 and CL4 (Table 3) also placed these two commercial varieties in the lower graph area (Figure 5A).

In the baby stage, L11 was located on the right (Figure 5A) due to its high levels of Car (Figure 3B), AsA, and Phe (Figure 4A,B). CL4 also presented good Car and AsA levels. The lowest Car, AsA, and Phe concentrations in the experiment observed for lettuce CL3 placed this commercial variety further to the left in the plot. For the second PC, these 3 varieties together with L2 were placed at the top of the plot due to their significant concentrations of minerals (Ca, K, and Fe, Table 3), and CL3 also presented the lowest dry biomass percentage in the baby stage (Figure 2). In contrast, the high DW value of CL5, together with a low mineral content, meant that this commercial variety was located at the bottom of the plot.

L11 in the adult stage once again presented the highest AsA, Phe, and DPPH levels, and it was located further to the right in Figure 5C, followed by CL4 and L2, which displayed significant levels for these traits. Unlike the other 2 stages, the variety further to the right in the adult format was CL5, which presented the lowest AsA content throughout the experiment, together with low Phe and DPPH levels (Figure 4A–C). According to the second PC, the most remarkable variety was CL4 (top of the plot), which occupied first and second places regarding the Phe and Car contents. L1 (bottom of the plot) also presented one of the lowest Car concentrations during the experiment in the adult stage.

### 3.5. Correlation between Quality Compounds

Correlation analyses were carried out to estimate the relation between the most important quality traits in the three development stages (Table 5).

Among the microgreens, the pairwise coefficients showed a positive correlation and a statistical significance for 6 out of the 36 studied pairs of traits. The most representative positive relations were observed between Phe and DPPH (r = 0.721) and Car and AsA (r = 0.505). Statistically significant negative correlations were also observed for 4 of the 45 studied pairs of traits. The closest negative relations were observed for K vs. Fe concentrations (r = −0.604) and K vs. Car (r = −0.494).

In the baby stage, the number of positive correlations rose to 13 and the strongest coefficients were observed for AsA vs. Car, AsA vs. Ant, and Asa vs. Phe (r between 0.615 and 0.697).

All significant pairwise coefficients in the adult lettuces showed positive correlations, including 17 of the 36 studied pairs of traits. The most representative relations were observed between the several Ant, AsA, Phe, and DPPH combinations, with the highest values observed for the pairs AsA vs. Phe, AsA vs. DPPH, and Phe vs. DPPH (r between 0.867 and 0.703). Important relations (r between 0.519 and 0.799) were observed for minerals (Ca and Fe) and several quality traits (Ant, AsA, Phe, and DPPH).

## 4. Discussion

Although lettuce is particularly known for its high water percentage and low calorie content [5], as it is generally consumed and marketed whole and raw [26], more nutrients are preserved than in other cooked or processed vegetables. Thus, its nutritional benefits related to its dietary fiber, mineral and vitamin contents, and several bioactive compounds, such as carotenoids and phenolic compounds, remain [5].

As several authors, such as Mou [27], Kim et al. [5], Kiriacou et al. [28,29], and Wojdylo et al. [30] have observed, the nutrient content of lettuce is determined by genetics, environmental influence, genotype–environment interactions, and plants’ harvest stage. Microgreens and baby lettuces may have significantly higher levels of vitamins, minerals, and other health beneficial phytonutrients than mature leaves. For these reasons, these types of seedlings are now appreciated as functional foods [31,32,33,34,35]. Seeds are a source of proteins, carbohydrates, and sometimes fats but not vitamins [36]. However, germination and embryo growth promote intense metabolic activity in seeds, in which several chemical reactions take place, including enzyme synthesis. Most carbohydrates and fats are reused in the synthesis of vitamins, sugars, proteins, and mineral salts [36]. Because of these processes, seedlings are considered as functional foods with substantial health-promoting properties [33]. This statement is reflected in our study because the studied varieties’ antioxidant capacity, including the main antioxidant compounds, such as ascorbic acid and phenols, showed a clear pattern that was repeated in all cultivars that microgreens and baby greens presented higher antioxidant properties than adult plants.

Phenols and ascorbic acid serve as scavengers of reactive oxygen species to protect young expanding leaves that are prone to light damage [17]. Phenolic compounds also seem to influence the sensory qualities of microgreens. In this regard, Xiao et al. [37] reported that the total Phe concentration correlates with the overall eating quality and several sensory qualities. According to Manjula et al., leafy vegetable microgreens present 2- to 5-fold more nutrients than mature leaves from adult vegetables [38]. In our study, this tendency was proven because the Phe content was almost 5-fold higher in seedlings than in adult lettuces regardless of the variability observed among varieties. Our results about adult lettuces are in line with Liu et al. [39], Mmapholo et al. [26], Huang et al. [40], and Kim et al. [5], who claimed that red-leaf adult cultivars have greater total Phe contents. This finding was observed in our trial as highlighted by the varieties CL4 and L11. These results are higher than the values reported by several authors for red lettuce cultivars [5,26,27,41] and are also higher than the values obtained from vegetables and fruit with known for elevated Phe contents, such as spinach (2.69 mg g^−1^ FW) [42], red onion (2.53–3.11 mg g^−1^ FW) [43], strawberry (3.64 mg g^−1^ FW) [43], plum (3.04 mg g^−1^ FW), and blueberry (4.25 mg g^−1^ FW) [44]. In the initial development stages, the Phe content of the varieties CL4 and L11 is similar to that of other varieties a priori qualified as less reddish, such as L3 and L5 in the baby green stage. However, compared to the Phe content in many other cultivar microgreens, such as beetroot (166 mg g^−1^ FW) or amaranth (586 mg g^−1^ FW) [30], lettuce microgreen cultivation is not remarkable. This fact does not seem to affect consumer choice because lettuce and carrot are some of the most preferred microgreens, followed by green peas, red amaranth, and finger millet [45].

Likewise, significant differences were detected when determining the total vitamin C concentration of the varieties. In addition, the AsA content was 41% higher in the seedling stages than in adult lettuces. Similar to phenols, the red varieties CL4 and L11 stood out from the rest. Similarly, when comparing our results to those of other authors [27,46,47], the obtained values, especially those in landraces, regarding the vitamin C content were higher for our varieties to to equivalent ones in terms of the lettuce type, based mainly on the color or head structure, in other articles. These results are similar to the vitamin C values obtained for other species: peas (30.9 mg 100 g^−1^ FW), spinach (31.6 mg 100 g^−1^ FW), green beans (15.1 mg 100 g^−1^ FW) [48], grapefruit (39.0 mg 100 g^−1^ FW), banana (11.1 mg 100 g^−1^ FW), and mango (37.0 mg 100 g^−1^ FW) [49]. Even colored lettuce varieties show vitamin values similar to crops known for their high ascorbic contents, for example, orange (49.4 mg 100 g^−1^ FW), pepper (50.3 mg 100 g^−1^ FW) [50], mandarin (57.4 mg 100 g^−1^ FW), and blueberry (60.1 mg 100 g^−1^ FW) [44]. Similarly, when comparing the values obtained for the microgreens and baby greens, even the values obtained in microgreens and baby greens equaled those obtained for broccoli (77.1 mg 100 g^−1^ FW) [48] and strawberry (77.3 mg 100 g^−1^ FW) [49]. These values are also comparable to those detected in other microgreen species, such as carrot (65.6 mg 100 g^−1^ FW), onion (29.9 mg 100 g^−1^ FW), spinach (71.2 mg 100 g^−1^ FW), and radish (88.5 mg 100 g^−1^ FW) [38].

The antioxidant capacity followed the same pattern as the phenolic compounds and vitamin C contents, and was more prominent in the microgreen and baby green stages. However, as no quantification was carried out, we were unable to perform a comparison with other crops, and only a comparison of the varieties under study was feasible. As previously mentioned, landrace L11 stood out in the adult stage. These data imply that both phenols and vitamin C are determinants of the generally increased antioxidant capacity of this crop because the varieties that stood out for these nutraceutical compounds tended to have a much higher antioxidant capacity.

Similarly, some of the analyzed pigments are also apparently involved in the total antioxidant capacity, especially anthocyanins, the content of which is high in red-colored lettuce [51,52,53]. Moreover, several authors, such as Llorach et al. [41], Baslam et al. [54], and Kim et al. [5], have claimed that red pigmentation is indicative of the total Ant and Phe content, which also corroborates the correlations found between these two parameters together with the total antioxidant capacity in baby and adult lettuces. Nevertheless, no correlations were found in microgreens, perhaps because at the time of the seedlings’ initial growth, metabolic activity intensifies after germination [36], and anthocyanins begin to be synthesized together with the other phenolic compounds, but their antioxidant properties are still irrelevant. In addition, a strong genetic component or environmental factors, such as light, may also affect the synthesis and activity of anthocyanins because the anthocyanins concentration does not seem to follow a clear pattern but varies between varieties in different ways.

The nutritional value of lettuce varies for different varieties and environmental conditions [5,29,36,55]. Among the environmental factors, light is one of the most important variables that affects phytochemical concentrations in plants. Light conditions influence the morpho-physiology of microgreens, together with the biosynthesis and accumulation of phytochemicals [56,57,58]. According to Mou and Ryder [10], the lower nutritional value of some varieties is due to the marked enclosure of their leaves in the head structure as most of the edible head structure portion includes leaves that are not exposed to light. Moreover, the size and number of external leaves and the head type lead to differences in the light microenvironment between outer and inner leaves [54]. One clear example is the lower nutrient content of crisphead lettuce versus romaine types [59]. Of the varieties included in our study, only CL5, a commercial iceberg variety, was confirmed to have the lowest values of vitamin C, DPPH, Chl, DW, and Phe. It was undoubtedly the variety with the highest degree of leaf overlap in our study. However, the variety CL4, a variety with the most patent buds, stood out for its high proportion of nutraceutical compounds. This could be due to its characteristic purple color, which is indicative of high Ant and Car contents. Conversely, the Roman purple variety L11, which was the variety with the lowest degree of leaf overlap, stood out for almost every analyzed phytochemical. Indeed, the different degrees of leaf overlap between our varieties could have influenced the variability of the studied compounds in the adult stage. Likewise, as our analyses were carried out in different lettuce development stages, the nutritional quality pattern between varieties was not maintained as no head structure was present in the youngest stages (microgreens and baby). This meant that varieties were highlighted when microgreens were not necessarily the most outstanding in the baby leaf or adult stages. In this regard, as consumers, food nutritionists, and producers are showing more interest in the health-related effects of the products they eat [6], the information presented herein could be helpful to guide consumers in their diet choices.

As mentioned earlier, the anthocyanin synthesis rate appeared to be variety dependent as no firm pattern was observed for production throughout the development of the studied varieties. For microgreens, narrow variability was observed between varieties and the Ant content did not seem to be proportional to the color of these seedlings, which occurred in more advanced development stages. This implies that other pigments absorbed at the same wavelength as anthocyanins were synthesized on a large scale. This could interfere with color determination in microgreens. Some perfect examples of this statement are the varieties CL4 (with a completely red first true leaf) and L1 (completely green-colored seedlings). Landrace L1 had the highest Ant concentration in our study (65 µmol 100 g^−1^ FW) while CL4 matched the varieties with the lowest concentration (36.8 µmol 100 g^−1^ FW) in the microgreen stage. In the following development stages, the reddish plant coloration was in accordance with the measured anthocyanin concentration. The variety CL4 in the baby green stage (155 µmol 100 g^−1^ FW) and landrace L11 in the adult stage (70.65 µmol 100 g^−1^ FW) were highlighted and, thus confirm the theory that red lettuce coloration is indicative of Ant content [5,41,54,60].

This also supports the notion that the higher the Ant content, the greater the light exposure [60], which was favored by low degrees of leaf overlap (open lettuce vs. crisphead formats), in addition to longer exposure times (adult vs. early stages).

As previously reported, differences in the carotenoids content between lettuce types has been suggested to be related to the head structure because it is regulated by light [5,10]. In addition, the increase in these pigments is beneficial due to their antioxidant properties [26,61]. In the juvenile development stages, the positive correlation between carotenoids and phenols was irrelevant, but the trend was positive and became statistically significant when plants reached maturity. This indicates the contribution of these pigments to the total antioxidant activity. This event has also been observed in ginger [62] and palm oils [63]. In our study, the lack of a direct correlation between carotenes and the antioxidant capacity of lettuce in all the development stages could be due to the antioxidant role of carotenes not being as relevant as that of phenols, anthocyanins, or vitamin C. Regardless of the observed wide inter-varietal variability, the carotenoids content in microgreens was practically null while the highest values were observed for baby plants. According to Wojdylo et al. [30], these results are unexpected because he claims that microgreens contain high levels of carotenoids and chlorophylls, among others. This could indicate that the machinery used for carotenoid production is activated late during development. Once again, the need to know each variety and its optimum harvesting period to obtain the best nutritional benefit is highlighted. The highest Car values were observed for the varieties L11 (114.2 µg g^−1^ FW), CL4 (51.5 µg g^−1^ FW), and L10 (48.9 µg g^−1^ FW) in the baby stage. In particular, the value obtained for landrace L11 was comparable to other crops known for their high carotenoid contents, such as red peppers (63–130 μg g^−1^ FW) [64,65] and carrots (95.9 μg g^−1^ FW) [66].

As far as the Chl content is concerned, it has been shown to depend not only on light itself, but also on the quality of this resource [67]. Similarly, it has been demonstrated that Fe is responsible for the biosynthesis of this pigment, at least the water-soluble Fe fraction [68]. Fe-deficient plants are usually characterized by the development of marked chlorosis, which lowers both the chlorophyll and carotenoid concentrations [69]. However, in our study, we observed a negative relation between the Chl content and the total Fe concentration, which became more pronounced in the adult plant stage. When focusing on this development stage, the relation between Ant and Chl was also negative while that with Fe was positive. As this study did not use an Fe-deficient environment, and as all the varieties ranged within the optimal Fe concentration for this crop in the adult stage (0.41–2 mg 100 g ^−1^ FW [59]), this might indicate that reddish varieties have a greater capacity to absorb or accumulate Fe, likely thorough more efficient Fe acquisition or transport systems. This does not imply that greener ones are Fe deficient and are therefore not capable of producing Chls. One clear example of this statement would be CL2. This variety has completely green leaves and is one of the accessions with the highest Chl content in our study but was ranked last regarding the Fe concentration. Other factors could also affect this relation, such as the contents of other pigments, such as carotenes, or other minerals also related to chlorophyll synthesis, such as Mg, which is a structural constituent of chlorophylls [70].

For Ca, a similar trend to that observed for Fe was noted because significant relations between this mineral and other phytonutrients were detected in the adult stage. The Ca concentration was generally higher in microgreens. This finding coincides with Pinto et al. [9]. In our assay, once plants reached maturity, the concentration of this mineral appeared to be correlated with the content of the main antioxidant compounds. The benefits derived from Ca application are well known, especially in postharvest activities, maintaining cell turgor and tissue firmness, delaying the catabolism of membrane lipids [71], and reducing fruit browning [72] by prolonging the storage life of fresh fruit [73]. Similarly, there is evidence that Ca promotes anthocyanin synthesis in vitro [74]. Although these facts may support the relation between Ca and the major antioxidants in lettuce, Ca can be obtained from the substrate, and plays an essential role in plant development and overall plant health. In lettuce leaf tissue, an increase in Ca enhances both the photosynthetic capacity and chlorophyll synthesis [75,76], which implies greater primary production of glucose and fructose from photosynthesis [76].

Finally, K is one of three major nutrients required for normal plant growth, and is involved in plant photosynthesis, transpiration, growth, and development [75]. Several studies have suggested that plant growth and yield are strongly affected by substrate K availability [77,78]. Our study did not carry out a comparative study between different substrate types. This only confirms that some varietal genetic differences enable them to capture and/or retain a certain K concentration What we were able to verify was the tendency to accumulate this mineral in lettuce leaves throughout development, which was explained by Kyriacou et al. [34], Pinto et al. [9], and el El-Nakhel et al. [17]. The K concentration in mature leaves was much higher than in microgreens for almost all varieties under study.

## 5. Conclusions

Considering lettuce’s fraction of functional compounds and its high consumption rate, it constitutes a very interesting source of nutrients (minerals and functional compounds). The results of the present study show that the nutrient content depends on the lettuce type, color, and development stage. Comparative nutrient data of several popularly consumed lettuce cultivars were obtained and analyzed, which will help consumers to choose foods with higher nutritional value.

Of all the studied varieties, landrace L11 was significant in all the studied stages and in almost all the analyzed parameters. This indicates the significantly high potential of this traditional reddish variety, and is of interest to consumers because of its attractive color. However, except for the commercial variety CL4, for the other studied cases, it is advisable to promote the production of varieties in stages other than the adult stage. Some examples of this include: CL5, which was found to be the most deficient in phytonutrients upon maturity but had high DPPH and AsA contents in microgreens; landrace L5, a variety that was not particularly significant in the analysis, and was observed to have significant total DPPH and Chl, Car, and AsA contents in the baby green stage. In turn, this underscores the idea of the significant existing but barely exploited variability of traditional varieties.

## Figures and Tables

**Table 1:** Abbreviation, origin, identification, and short phenotypic description of the 11 lettuce varieties used in this study. The plant material from local landraces was provided by the: (1) Valencian Institute for the Conservation and Improvement of Agrobiodiversity (COMAV, Valencia, Spain); (2) Valencian Institute for Agricultural Research (IVIA, Moncada, Spain).

Abbreviation Code	Origin	Identification	Plant Description
CL1	Commercial	Romaine lettuce long mule ear (Battle) ^a^	Dark green. Elongated shape. Compact and narrow head, barely prominent.
CL2	Commercial	Romaine lettuce from the gardeners (Vilmorín) ^a^	Green-yellowish. Elongated shape. Compact and narrow head, barely prominent.
CL3	Commercial	Wonder summer (Battle) ^a^	Green with reddish shades. Remarkable width in relation to height. Compact, rounded and quite prominent head.
CL4	Commercial	Marvel of Four Seasons Butterhead (Battle) ^a^	Dark green with reddish shades; the red is prominent at the edges. Round shape. Quite rounded shape. Full-sized head.
CL5	Commercial	Batavia, iceberg type (Battle) ^a^	Not very intense green. Rounded shape. Full-sized head.
L1	Local landrace	BGV5721 ^b,1^	Dark green. Pink shades near the principal stem. Elongated shape. Compact and narrow head, barely prominent.
L2	Local landrace	BGV5722 ^b,1^	Green-yellowish. Round shape. Full-sized head.
L3	Local landrace	BGV5723 ^b,1^	Green-yellowish. Remarkable width in relation to height. Head not appreciated.
L5	Local landrace	BGV5736 ^b,1^	Dark green with reddish shades. Elongated shape. Compact and narrow head, quite prominent.
L10	Local landrace	L-10 ^b,2^	Dark green. Elongated shape. Compact and narrow head, barely prominent.
L11	Local landrace	L-11 ^b,2^	Dark red, almost purple. Remarkable width in relation to height. Head not appreciated.

^a^ Commercial name (company), ^b^ Genbank code.

**Table 2:** Variation parameters for the quality traits in the 11 lettuce varieties evaluated in the 3 development stages (microgreen, baby, adult). Statistics were performed per stage. Values represent the mean, range, coefficient of variation (CV, %), F-ratio, and significance (***, **, * indicate significance at *p* < 0.001, *p* < 0.01, *p* < 0.05) for the quality traits. DW: Dry weight; Chl: Chlorophylls; Car: Carotenes; Ant: Anthocyanins; AsA: Ascorbic Acid; Phe: Phenols; DPPH: Antioxidant capacity; Ca: Calcium; K: Potassium; Fe: Iron.

	Unit/Scale	Mean	Range	CV (%)	F-Ratio
Microgreen					
DW	%	7.53 ± 0.59	5.81–8.84 ***	7.84	4.81
Chl	µg g^−1^ FW	393.77 ± 92.38	200.89–647.87 ***	23.46	29.45
Car	µg g^−1^ FW	3.70 ± 9.62	0–43.48 ***	259.62	70.10
Ant	µmol 100 g^−1^ FW	50.67 ± 10.29	36.12–72.34 ***	20.31	20.55
AsA	mg 100 g^−1^ FW	61.05 ± 11.61	34.26–87.01 ***	19.02	54.38
Phe	mg g^−1^ DW	18.69 ± 5.23	7.43–28.78 ***	28.01	24.3
DPPH	%	71.52 ± 21.17	12.65–88.46 ***	29.61	32.08
Ca	mg g^−1^ DW	9.76 ± 1.75	6.61–12.81 ***	17.96	22.48
K	mg g^−1^ DW	48.23 ± 3.93	39.54–60.08 **	8.15	2.97
Fe	µg g^−1^ DW	225.08 ± 60.74	68.1–362.18 ***	26.99	132.5
Baby					
DW	%	10.14 ± 1.14	7.65–12.74 ***	11.26	13.03
Chl	µg g^−1^ FW	502.85 ± 65.60	339.05–606.61 ***	13.05	14.63
Car	µg g^−1^ FW	36.76 ± 28.89	1.87–126.98 ***	78.6	36.93
Ant	µmol 100 g^−1^ FW	56.16 ± 29.88	26.46–141.96 ***	56.19	296.27
AsA	mg 100 g^−1^ FW	58.35 ± 11.19	39.25–86.49 ***	19.18	34.83
Phe	mg g^−1^ DW	18.43 ± 8.49	5.58–36.11 ***	46.06	45.24
DPPH	%	71.07 ± 13.46	32.61–87.02 ***	18.93	23.79
Ca	mg g^−1^ DW	7.23 ± 1.28	5.39–10.07 ***	17.65	7.39
K	mg g^−1^ DW	28.18 ± 3.60	22.21–36.82 *	12.78	2.53
Fe	µg g^−1^ DW	104.64 ± 18.07	71.24–146.55 ***	17.27	4.28
Adult					
DW	%	6.48 ± 1.46	3.83–13.25 ***	22.5	6.84
Chl	µg g^−1^ FW	434.36 ± 46.61	359.85–564.58 ***	10.73	7.88
Car	µg g^−1^ FW	11.11 ± 11.01	0–39.73 ***	99.10	35.03
Ant	µmol 100 g^−1^ FW	47.63 ± 10.50	29.15–71.76 ***	22.08	12.65
AsA	mg 100 g^−1^ FW	34.45 ± 14.33	13.44–64.2 ***	41.58	88.68
Phe	mg g^−1^ DW	3.91 ± 2.50	0.56–9.38 ***	63.89	49.97
DPPH	%	15.88 ± 9.55	3.58–39.41 ***	60.14	19.21
Ca	mg g^−1^ DW	8.87 ± 1.79	6.13–13.79 ***	20.18	23.04
K	mg g^−1^ DW	59.89 ± 7.66	44.36–75.96 ***	12.80	11.55
Fe	µg g^−1^ DW	171.44 ± 114.32	74.51–514.25 ***	66.68	131.98

**Table 3:** The calcium (Ca), potassium (K), and iron (Fe) concentrations of 11 lettuce varieties evaluated in 3 development stages (microgreen, baby, adult). Values are the mean ± SE of four replicates per variety. The means were subjected to a one-way ANOVA analysis. Different capital and lowercase letters indicate significant differences between varieties and development stages, respectively, at *p* < 0.05 using the LSD test. DW: Dry weight.

Variety	State	Ca (mg g^−1^ DW)		K (mg g^−1^ DW)		Fe (µg g^−1^ DW)	
CL1	Microgreen	8.76 ± 0.87	DEFa	48.06 ± 1.26	Bb	230.48 ± 20.05	CDa
	Baby	5.64 ± 0.16	Cc	26.47 ± 3.03	CDc	119.34 ± 16.77	Ab
	Adult	7.21 ± 0.03	Eb	58.13 ± 2.63	Ca	108.80 ± 7.19	DEb
CL2	Microgreen	8.66 ± 0.54	DEFa	45.85 ± 4.56	BCb	230.51 ± 2.06	CDa
	Baby	6.65 ± 0.82	BCb	25.37 ± 3.35	Dc	92.90 ± 9.12	Cb
	Adult	7.19 ± 0.17	Eb	68.73 ± 4.84	Aa	84.30 ± 10.40	Eb
CL3	Microgreen	10.91 ± 1.35	Ba	53.59 ± 4.36	Ab	77.74 ± 10.88	Gb
	Baby	7.93 ± 0.82	Ab	32.53 ± 2.61	Ac	114.69 ± 8.51	ABb
	Adult	8.76 ± 0.65	CDb	66.02 ± 4.46	ABa	218.88 ± 38.54	Ba
CL4	Microgreen	9.66 ± 1.29	Ca	47.14 ± 2.89	Bb	155.56 ± 4.73	Fb
	Baby	7.76 ± 0.47	Ab	31.86 ± 1.87	ABc	118.73 ± 6.45	Ab
	Adult	11.07 ± 0.70	Ba	57.91 ± 6.47	Ca	193.38 ± 12.79	Ba
CL5	Microgreen	8.21 ± 0.23	EFa	48.92 ± 3.79	ABa	255.54 ± 12.62	Ba
	Baby	5.88 ± 0.46	Cc	26.46 ± 2.02	CDb	94.77 ± 16.44	Cb
	Adult	6.82 ± 0.61	Eb	46.21 ± 1.68	Da	110.06 ± 22.83	DEb
L1	Microgreen	7.72 ± 0.58	Fa	48.73 ± 4.69	Bb	216.87 ± 8.87	DEa
	Baby	6.23 ± 0.55	Cb	27.89 ± 1.65	BCDc	91.43 ± 13.50	Cb
	Adult	7.51 ± 0.83	Ea	66.53 ± 5.29	ABa	123.30 ± 10.28	CDab
L2	Microgreen	12.37 ± 0.15	Aa	47.69 ± 0.86	Ba	344.91 ± 13.64	Aa
	Baby	8.89 ± 1.02	Ab	30.25 ± 5.41	ABCb	119.75 ± 20.78	Aa
	Adult	9.47 ± 0.71	CDb	50.63 ± 1.14	Da	119.34 ± 3.73	Db
L3	Microgreen	7.77 ± 0.79	EFab	49.18 ± 3.43	ABb	244.98 ± 3.66	BCDa
	Baby	6.50 ± 1.03	Cb	28.21 ± 5.60	ABCDc	98.21 ± 5.05	BCb
	Adult	8.78 ± 0.73	CDa	66.10 ± 4.57	ABa	100.87 ± 13.03	DEb
L5	Microgreen	12.38 ± 0.23	Aa	49.73 ± 2.88	ABb	222.59 ± 9.10	DEa
	Baby	8.58 ± 1.18	Ab	25.48 ± 0.46	Dc	89.74 ± 13.70	Cb
	Adult	9.70 ± 1.01	Cb	59.69 ± 4.42	Ca	153.94 ± 27.85	Cab
L10	Microgreen	10.66 ± 0.15	BCa	49.57 ± 1.33	ABb	208.97 ± 2.41	Eab
	Baby	7.77 ± 0.68	ABb	26.68 ± 1.41	CDc	88.93 ± 12.13	Cb
	Adult	8.65 ± 0.92	Db	56.93 ± 4.46	Ca	152.15 ± 19.59	Cb
L11	Microgreen	10.28 ± 0.12	BCb	42.13 ± 2.81	Cb	236.34 ± 9.73	BCDb
	Baby	8.07 ± 1.09	Ac	28.77 ± 2.17	ABCDc	122.49 ± 17.73	Ab
	Adult	12.41 ± 0.92	Aa	61.91 ± 1.62	BCa	493.47 ± 23.01	Aa

**Table 4:** Correlation coefficients for the quality traits of the 3 first principal components, eigenvalue, and the relative and cumulative proportions of the total variance explained by these components, in 11 lettuce varieties evaluated in 3 development stages (microgreen, baby, adult). DW: Dry weight; Chl: Chlorophylls; Car: Carotenoids; Ant: Anthocyanins; AsA: Ascorbic Acid; Phe: Phenols; DPPH: Antioxidant capacity; Ca: Calcium; K: Potassium; Fe: Iron.

	First PC	Second PC	Third PC
Microgreen			
DW	0.355	−0.230	
Chl a + b	0.203	−0.557	
Car	0.334	−0.294	0.213
Ant			−0.665
AsA	0.419		
Phe	0.366	0.352	
DPPH	0.373	0.346	
Ca		0.270	0.653
K	−0.409		−0.182
Fe	0.295	0.435	
Eigenvalue	4.70	2.28	1.35
Variance explained (%)	46.99	22.80	13.49
Cumulative variance explained (%)	46.99	69.79	83.28
Baby			
DW	0.201	−0.471	
Chl a + b	0.292		0.509
Car	0.459		0.221
Ant	0.335		−0.545
AsA	0.510		
Phe	0.413	0.167	0.180
DPPH	0.284	−0.269	−0.466
Ca		0.403	0.292
K		0.553	−0.153
Fe	0.169	0.436	−0.179
Eigenvalue	3.46	2.64	1.39
Variance explained (%)	34.60	26.43	13.90
Cumulative variance explained (%)	34.60	61.02	74.93
Adult			
DW	0.328		−0.326
Chl a + b	−0.360		
Car		0.593	−0.415
Ant	0.331	−0.270	0.242
AsA	0.396	0.157	
Phe	0.369	0.334	
DPPH	0.406		
Ca	0.248	−0.428	−0.218
K		0.388	0.718
Fe	0.313	−0.289	0.283
Eigenvalue	5.09	1.44	1.07
Variance explained (%)	50.88	14.40	10.73
Cumulative variance explained (%)	50.88	65.27	76.00

**Table 5:** Linear correlation coefficient (r) and its significance of the quality traits in the 11 lettuce varieties evaluated in 3 development stages (microgreen, baby, adult). ***, **, * indicate significance at *p* < 0.001, *p* < 0.01, *p* < 0.05 for r. Chl: Chlorophylls; Car: Carotenoids; Ant: Anthocyanins; AsA: Ascorbic Acid; Phe: Phenols; DPPH: Antioxidant capacity; Ca: Calcium; K: Potassium; Fe: Iron.

Microgreen									
	Chl a + b	Car	Ant	AsA	Phe	DPPH	Ca	K	Fe
Chl a + b		0.4143 *	−0.0364	0.3928 *	0.0747	0.0346	−0.117	−0.119	−0.0901
Car			0.0732	0.5045 **	0.1708	0.2554	−0.1941	−0.4941 **	0.2132
Ant				0.0124	0.2169	0.1641	−0.4458 **	−0.1202	0.2338
AsA					0.3244 *	0.4498 **	−0.1398	−0.3722 *	0.1853
Phe						0.7212 ***	0.262	−0.2496	0.2812
DPPH							0.1729	−0.1802	0.1598
Ca								0.0285	0.0943
K									−0.604 ***
Fe									
**Baby**									
	**Chl** **a** **+** **b**	**Car**	**Ant**	**AsA**	**Phe**	**DPPH**	**Ca**	**K**	**Fe**
Chl a + b		0.5116 **	0.2391	0.3653 *	0.27	0.0557	0.0786	0.0099	0.0448
Car			0.271	0.6154 ***	0.4764 **	0.2258	0.1565	−0.0187	0.4546 **
Ant				0.6243 ***	0.2022	0.4963 ***	−0.0116	0.1843	0.116
AsA					0.6974 ***	0.4557 **	0.1692	−0.0295	0.3639 *
Phe						0.184	0.3394 *	0.0149	0.3192 *
DPPH							−0.1262	−0.2486	0.0874
Ca								0.4761 **	0.1332
K									0.2664
Fe									
**Adult**									
	**Chl** **a** **+** **b**	**Car**	**Ant**	**AsA**	**Phe**	**DPPH**	**Ca**	**K**	**Fe**
Chl a + b		−0.3158	0.3714 *	0.2159	0.1143	0.2218	0.0939	0.419 **	−0.0149
Car			0.15	0.0572	0.2014	0.0254	0.147	−0.0818	0.0858
Ant				0.5291 ***	0.4224 **	0.5695 ***	0.5614 ***	0.2667	0.584 ***
AsA					0.8672 ***	0.7029 ***	0.7997 ***	0.2131	0.5939 ***
Phe						0.7243 ***	0.6467 ***	0.1911	0.5194 ***
DPPH							0.5732 ***	0.0936	0.5226 ***
Ca								0.0815	0.7591 ***
K									0.0814
Fe									

## Data Availability

The datasets generated for this study are available on request to the corresponding author.

## Supplementary Materials

The following are available online at https://www.mdpi.com/article/10.3390/foods11030423/s1, Table S1: The sodium (Na), magnesium (Mg), phosphorus (P), sulphur (S), Zinc (Zn), manganese (Mn) and silicon (Si) concentrations in the collection of the 11 lettuce varieties evaluated in the three development stages (microgreen, baby, adult). Values are the mean±SE of four replicates per variety. The means are subjected to a one-way ANOVA analysis. Different capital and lowercase letters indicate significant differences between varieties and development stages, respectively, at *p* < 0.05 using the LSD test. DW: Dry weight.
